# Editorial: In Celebration of Women in Science: Biological Modeling and Simulation

**DOI:** 10.3389/fmolb.2023.1175325

**Published:** 2023-04-27

**Authors:** Burcu Aykac Fas, Sophie Sacquin-Mora, Elena Papaleo

**Affiliations:** ^1^ Laboratoire de Biochimie Théorique UPR 9080 CNRS, Institut de Biologie Physico-Chimique, Paris, France; ^2^ Cancer Structural Biology, Danish Cancer Society Research Center, Copenhagen, Denmark; ^3^ Cancer Systems Biology, Section for Bioinformatics, Department of Health and Technology, Technical University of Denmark, Lyngby, Denmark

**Keywords:** modeling, molecular dynamics (MD) simulations, quantum mechanics, monte carlo, brownian dynamics, integrative computational-experimental approaches, biomolecules, biosensors

Although the percentage of women choosing Science, Technology, Engineering, and Mathematics (STEM) fields changes over time, the positions occupied by women within them still tend to be more junior compared to those of men. In this context, each initiative to raise community awareness of this disparity no matter how small, will likely make a difference for the next generations.

After decades of invisibilization, it is essential to acknowledge society’s past failures in recognizing women’s contributions to STEM. Biases that are difficult to remove from societal thinking and stereotypes often discourage women from following a career track in science-related fields; the field of Molecular Modeling is especially affected by this and is highly male-dominated.

In the study by Bonham and Stefan (2017) on women’s underrepresentation in computational biology, primary publications from 1997 to 2014 were scanned in biology, computer science, and computational biology. The percentages of women in the first and last author positions were found to be around 25% and 38%, respectively, for the biology field, and 4-6 percentage points lower, consistent across all authorship positions for the computational biology field. These numbers are consistent with the work of Holloway and McGaughey (2018), who estimated the female representation in computational chemistry to be approximately 25%. As an interdisciplinary STEM field, computational biology was found to lie between its parent fields as long as the female representation continues. It should also be noted that in these publications, if the person leading the study was female, there were more women in other authorship positions, pointing out to women mentors and principal investigators (PI), attracting more female collaborators (Bonham and Stefan, 2017). The implication is that having more female leaders can improve the global gender balance in STEM. This goal should be pursued not only on moral and social grounds but also because of its benefits to the scientific world.

Frontiers in Molecular Biosciences—Biological Modeling and Simulation had the ambition of contributing to the awareness of gender equality through this Research Topic. Here, we showcase the success of several female researchers at any step in their scientific careers in producing valuable research findings in the field of Modeling and Simulations. It is relevant to note that among the eight contributions (seven original research articles and one review) accepted for publication in this Research Topic, several different domains were covered: quantum mechanical models, molecular dynamics simulations, approaches for the integration of experimental biophysical data and molecular modeling, along methods based on network theory to understand protein structures. The articles covered molecular modeling applications across various fields, such as nanoparticles, sensors, proteins, DNA lesions, and metal clusters, attesting to the multidisciplinary nature of these methodologies.

(Martin et al.) focused on the dynamic nature of protein-protein interfaces to provide a complete picture of protein-protein complexes, considering that they visit distinct interface substates beyond a specific interface size. They studied the interfaces using molecular dynamics (MD) simulations, highlighting the crucial role of water in the binding and strength of the interactions in terms of the number of released water molecules and the number of contacts mediated by water molecules at the interface. The number of water-mediated contacts observed in the simulations of the complexes suggests that in protein-protein docking, the presence of water molecules at the interface may open a new route to predict them. (Koder Hamid et al.) investigated the possible structural changes of a surface-active intrinsically disordered protein (IDP) induced by adsorption while evaluating seven different force field and water model combinations and comparing them with experiments. They demonstrated the substantial influence of the choice of force field and water model in MD simulations when studying IDPs.

Owing to their low diffraction power and the complexity of sample delivery, X-ray free-electron laser (XFEL) experiments and data analyses are challenging for biological systems. The hybrid method (Asi et al.) proposed can be applied to single-particle XFEL diffraction patterns to obtain plausible 3D structural models by optimizing a known structure to maximize the similarity between the target XFEL and the simulated diffraction patterns from candidate models using Monte Carlo (MC) sampling. The proposed method can serve as an alternative approach for studying the dynamics/conformational transitions of biomolecules in XFEL experiments to seek initial models for 3D reconstruction algorithms and structure determination via 3D reconstruction from a large dataset.

8-Oxoguanine oxidatively generated lesions on RNA strands constitute a hallmark marker of oxidative stress in the cell, and recognition of these damaged RNA strands by poly-C binding protein 1 (PCBP1) triggers a signaling pathway that leads to cell apoptosis. The study by (Gillet et al.) investigated the molecular mechanism of PCBP1 to recognize a damaged RNA strand using all-atom MD simulations. Their results highlighted an allosteric mechanism by comparing RNA and protein behaviors for sequences with six different damage profiles. The GCAT network tool developed by (Pacini et al.) considers the asymmetry of mutational changes and allows visualization of the mutational influences of amino acid positions on one another. It was built by comparing the position neighborhoods in the amino acid network of the wild-type and mutant forms of the proteins. GCAT opens up several perspectives for improving the understanding of protein variants, and their role in disease onset and drug treatment efficiency by addressing how positions cope with mutations, how to classify position tolerances, and the need for corrections.

(Dutta et al.) have integrated computational and experimental designs of a rationally designed gold-functionalized biosensor capable of capturing a target protein using a biotin-streptavidin pair as a proof-of-concept. They employed atomistic simulations at multiple levels of theory, combining docking by Brownian dynamics and kinetics and classical MD. They provided guidelines on how to exploit the microscopic parameters obtained from simulations to guide the design of biosensors with enhanced sensitivity. The study by (Moubarak et al.) addressed the structure of the active site of [ZnFe] sulerythrin (SulE) isolated from a strictly aerobic archaeon. The function of SulE was investigated using a combination of structural and electronic analyses based on quantum mechanical (QM) calculations. Density functional theory (DFT) computational investigations of the active site geometry of [ZnFe] SulE based on available structural data shed light on the assumed electronic and structural states of the enzyme.

A review article by (Bini et al.) discusses the exploitation of colloid science concepts to better understand and predict the phase behavior of functionalized nanoparticles and/or protein–nanoparticle mixtures. They perspectively revisit the phase behavior of isotropic potentials with increasing complexity, illustrating how the additional phases appear as specific features are added to the potential.
